# Inflammatory potential of diet and risk of cardiovascular disease or mortality: A meta-analysis

**DOI:** 10.1038/s41598-017-06455-x

**Published:** 2017-07-25

**Authors:** Xiaoming Zhong, Lin Guo, Lei Zhang, Yanming Li, Ruili He, Guanchang Cheng

**Affiliations:** Department of Cardiology, Huaihe Hospital of Henan University, Kaifeng, 475000 China

## Abstract

Inconsistent findings have reported on the inflammatory potential of diet and cardiovascular disease (CVD) and mortality risk. The aim of this meta-analysis was to investigate the association between the inflammatory potential of diet as estimated by the dietary inflammatory index (DII) score and CVD or mortality risk in the general population. A comprehensive literature search was conducted in PubMed and Embase databases through February 2017. All prospective observational studies assessing the association of inflammatory potential of diet as estimated by the DII score with CVD and all-cause, cancer-related, cardiovascular mortality risk were included. Nine prospective studies enrolling 134,067 subjects were identified. Meta-analyses showed that individuals with the highest category of DII (maximal pro-inflammatory) was associated with increased risk of all-cause mortality (hazard risk [HR] 1.22; 95% confidence interval [CI] 1.06–1.41), cardiovascular mortality (RR 1.24; 95% CI 1.01–1.51), cancer-related mortality (RR 1.28; 95% CI 1.04–1.58), and CVD (RR 1.32; 95% CI 1.09–1.60) than the lowest DII score. More pro-inflammatory diets, as estimated by the higher DII score are independently associated with an increased risk of all-cause, cardiovascular, cancer-related mortality, and CVD in the general population, highlighting low inflammatory potential diet may reduce mortality and CVD risk.

## Introduction

Cardiovascular disease (CVD) and cancer are the leading causes of death worldwide^1, 2^. Chronic inflammation is linked to CVD and certain types of cancers^3, 4^. Low-grade inflammation is frequently caused by lifestyle or environmental factors. Diets play an important role in regulating chronic inflammation^5, 6^. Human diets can be grouped into pro-inflammatory and anti-inflammatory components. The anti-inflammatory diets have been described on the basis of a Mediterranean dietary pattern^7, 8^. Individuals with a high consumption of vegetables, fruits, whole grains, nuts, seeds, healthy oils, and fish may have a low risk of inflammation-related diseases^9^. Adopting healthy dietary pattern may be the first step in reducing inflammation-associated chronic diseases.

In order to assess the inflammatory potential of an individual’s diet, a novel dietary inflammatory index (DII) score was developed to estimate the inflammatory potential of nutrients and foods in the context of a dietary pattern^10, 11^. The DII distinguishes dietary patterns on a continuum from the maximal pro-inflammatory to maximal anti-inflammatory. A higher DII score indicates a more pro-inflammatory diet and a lower DII score represents a more anti-inflammatory diet. Ever since then, several studies^12–21^ have examined the association between inflammatory potential of diet as measured by the DII score and mortality or CVD risk in the general population. However, these studies yielded the conflicting results^22^. Moreover, the magnitude of the risk estimates varied considerably. To the best of our knowledge, no previous a systematic review or meta-analysis has addressed this issue. Therefore, we conducted this meta-analysis of available prospective studies to examine the association of pro-inflammatory diets as estimated by the higher DII score with CVD and mortality risk in the general population.

## Results

### Literature search and study characteristics

**Figure 1:**
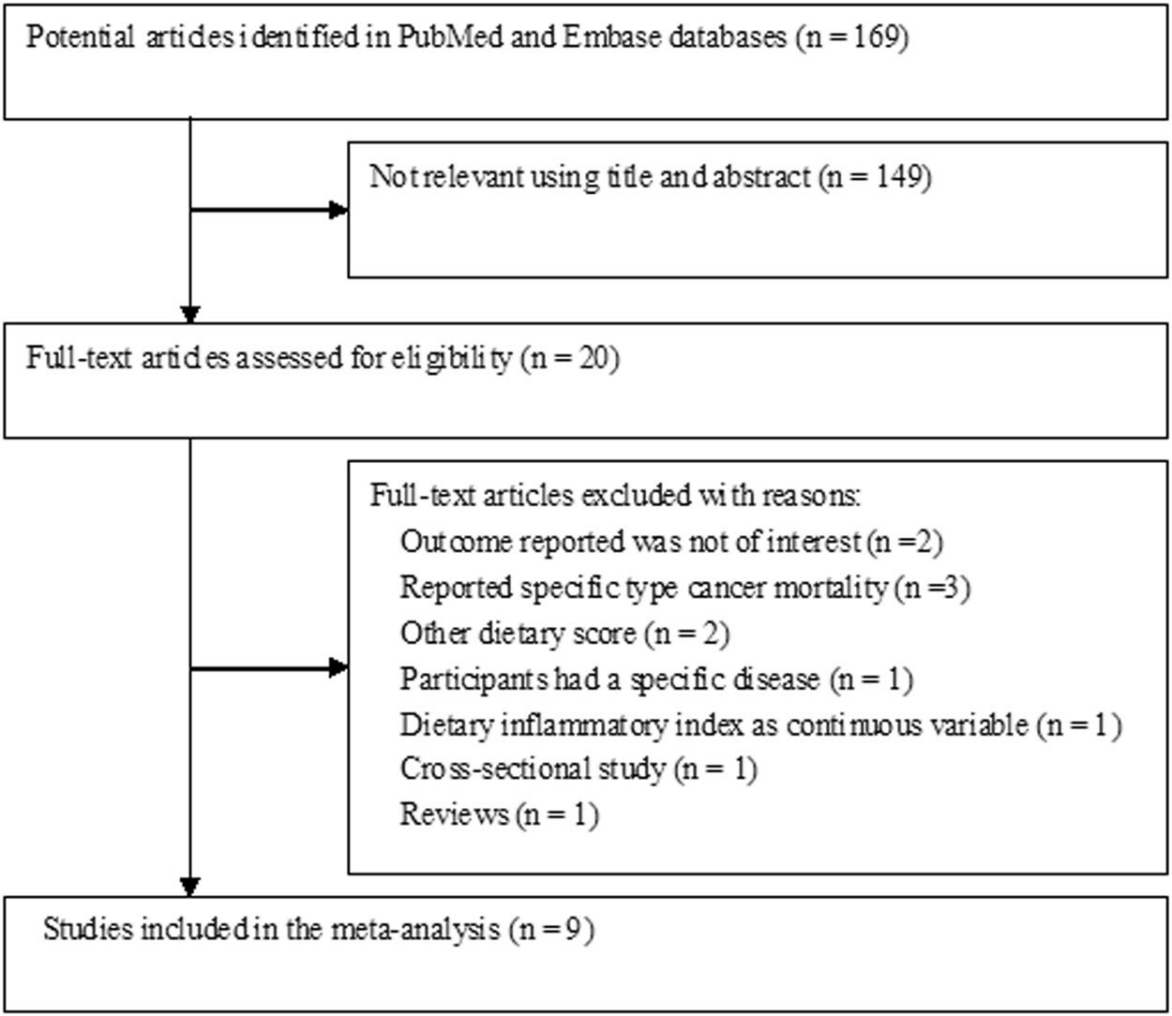
Flow chart of study selection process.

Briefly, a total of 179 relevant articles were identified with search terms. Of those, 20 were retrieved as full-text articles and 9 studies^12–20^ were finally included in the meta-analysis. The detailed study selection process is shown in Fig. 1. Table 1 summarizes the characteristics of the included studies. A total of 134,067 subjects were identified in these studies. The sample size of the included studies ranged from 1,363 to 37,525. Included studies were published between 2015 and 2017 and conducted in the United States^13, 15^, Spain^17, 18^, Sweden^12^, France^14, 20^, and Australia^19^. The follow-up duration ranged from 1.24 to 20.7 years. All nine studies achieved moderate to high methodological quality with a score from 6–8 stars and the mean NOS stars of the included studies was 7.0.

**Table 1:** Characteristics of studies included in meta-analysis.

Study/year	Country	Design	Subjects (% female)	Mean age or range (years)	DII score evaluation	DII score Comparison	Events/number RR or OR (95% CI)	Follow-up (years)	Adjustment for covariates	NOS Scores
Shivappa *et al*.^12^	Sweden	Prospective population- based cohort	33,747 (100)	46 ± 9.9	27 food items using FFQ	Quintile 5 vs. 1	Total death (7,095);	15	Age, energy intake, BMI, education, smoking status, PA, and alcohol intake.	7
							1.25 (1.07–1.47);			
							CV death (2,399);			
							1.26 (0.93–1.70)			
							All cancer (1,996);			
							1.25 (0.96–1.64)			
Shivappa *et al*.^13^	USA	Prospective cohort study	37,525 (100)	55–69	37 food items using FFQ	Quartile 4 vs. 1	Total death (17,793)	20.7	Age, BMI, smoking, pack-years of smoking, HRT use, education, prevalent DM, prevalent hypertension, prevalent heart disease, prevalent cancer, total energy intake	7
							1.08 (1.03–1.13);			
							CV death (6,528);			
							1.09 (1.01–1.18)			
							All cancer (5,044);			
							1.08 (0.99–1.18)			
Graffouillere *et al*.^14^	France	Prospective cohort study	8,089 (62.5)	49.1 ± 6.0	36 food items using 24-h dietary records	Tertile 3 vs. 1	Total death (207);	1.24	Age, sex, intervention group of the initial trial, number of 24-h dietary records, BMI, physical activity, smoking, education, family history of cancer or CVD in first-degree relatives, energy intake, and alcohol	6
							1.41 (0.97–2.04);			
							All cancer (123);			
							1.83 (1.12–2.99)			
Shivappa *et al*.^15^	USA	Prospective cohort study	12,438 (51.5)	47.2 ± 19.1	27 food items using 24-h dietary records	Tertile 3 vs. 1;	Total death (2,795);	11	Age, sex, race, DM, hypertension, PA, BMI, poverty index, and smoking	8
							1.34 (1.19–1.51);			
							CV death (1,223);			
							1.46 (1.18–1.81)			
							All cancer (615);			
							1.46 (1.10–1.96)			
O’Neil *et al*.^16^	Australia	Prospective cohort study	1,363 (0)	59.2 ± 19.2/59 ± 19.2	22 food items using FFQ	Positive vs. negative DII (cutoff value)	CVD (76)	5	Age, DM, SBP, DBP, smoking history, PA, waist circumference, and total daily energy consumption.	7
							2.00 (1.01–3.96);			
Ramallal *et al*.^17^	Spain	Prospective cohort study	18,974 (61)	38 ± 12	28 food items using FFQ	Quartile 4 vs. 1	CVD (117)	8.9	Age, sex, hypertension, dyslipidaemia, DM, smoking, family history of CVD, total energy intake, PA, BMI, education, other CVD, baseline special diet, snacking, average time sitting or spent watching television	8
							2.03 (1.06–3.88);			
Garcia-Arellano *et al*.^18^	Spain	Prospective cohort study	7,216 (57.4)	67 ± 6.2	32 food items using FFQ	Quartile 4 vs. 1	CVD (277)	4.7	Age, sex, overweight/obesity, waist-to-height ratio, total energy intake, smoking, DM, hypertension, dyslipidemia, family history of premature CVD, PA, education, and stratified by intervention and center	m
							1.73 (1.15–2.60)			
Vissers *et al*.^19^	Australia	Prospective cohort study	6,972 (100)	52 ± 1.0	25 food items using FFQ	Positive vs. negative DII (cutoff value)	CVD (335)	11	Age, energy, DM, hypertension, smoking, education, menopausal status, HRT use, PA and alcohol consumption	7
							1.03 (0.76–1.42)			
Neufcourt *et al*.^20^	France	Prospective cohort study	7,743 (42)	51.9 ± 4.7 (men) and 47.1 ± 6.6 (women)	36 food items using 24-h dietary records	Quartile 4 vs. 1	CVD (292)	11.4	Sex, energy intake, supplementation group, 24-h records, education, marital status, smoking, PA, and BMI	8
							1.15 (0.79–1.68);			

Abbreviations: OR, odds ratio; HR, hazard ratio; CVD, cardiovascular disease; CV, cardiovascular; DM, diabetes mellitus; SBP, systolic blood pressure; DBP, diastolic blood pressure; DII, dietary inflammatory index; FFQ, food frequency questionnaire; HRT, hormone replacement therapy; PA, physical activity.

### DII score and all-cause, cancer-related, cardiovascular mortality risk

**Figure 2:**
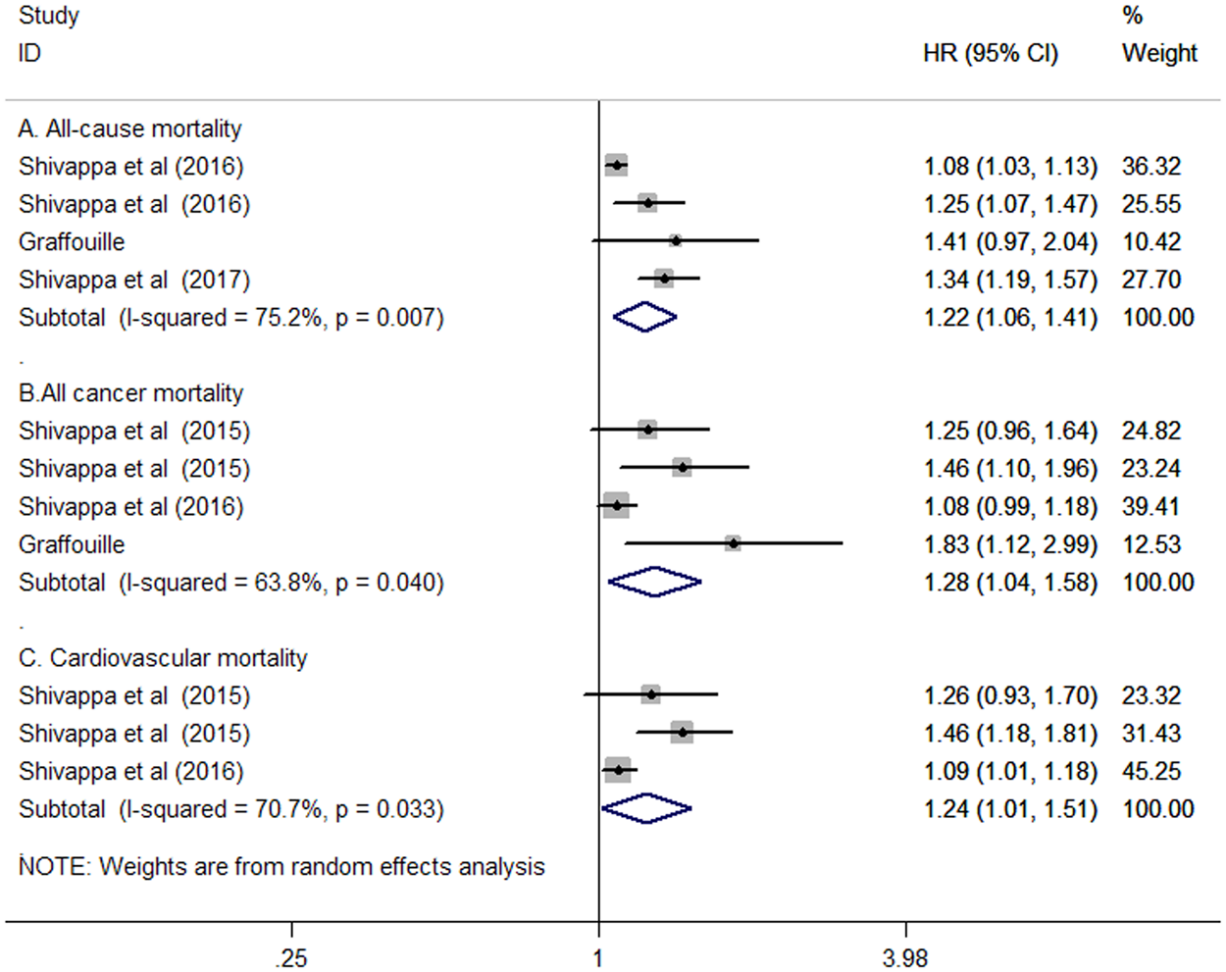
Forest plots showing HR with 95% CI of all-cause mortality (A), cancer-related mortality (B), and cardiovascular mortality (C) comparing the highest to the lowest dietary inflammatory index score.

Four studies^12–15^ involving 91,799 participants reported 27,890 all-cause mortality and 7,778 cancer-related mortality events. Three studies^12, 13, 15^ reported 10,150 cardiovascular mortality events from 83,710 participants. As shown in Fig. 2, when compared with those in the lowest DII category, participants in the highest category of DII (strongly pro-inflammatory) was associated with increased risk of all-cause mortality (HR 1.22; 95% CI 1.06–1.41; I^2^ = 75.2%, *p* = 0.007), cancer-related mortality (HR 1.28; 95% CI 1.04–1.58; I^2^ = 63.8%, *p* = 0.040), and cardiovascular mortality (HR 1.24; 95% CI 1.01–1.51; I^2^ = 70.7%, *p* = 0.033) in a random effect model.

### DII score and CVD risk

**Figure 3:**
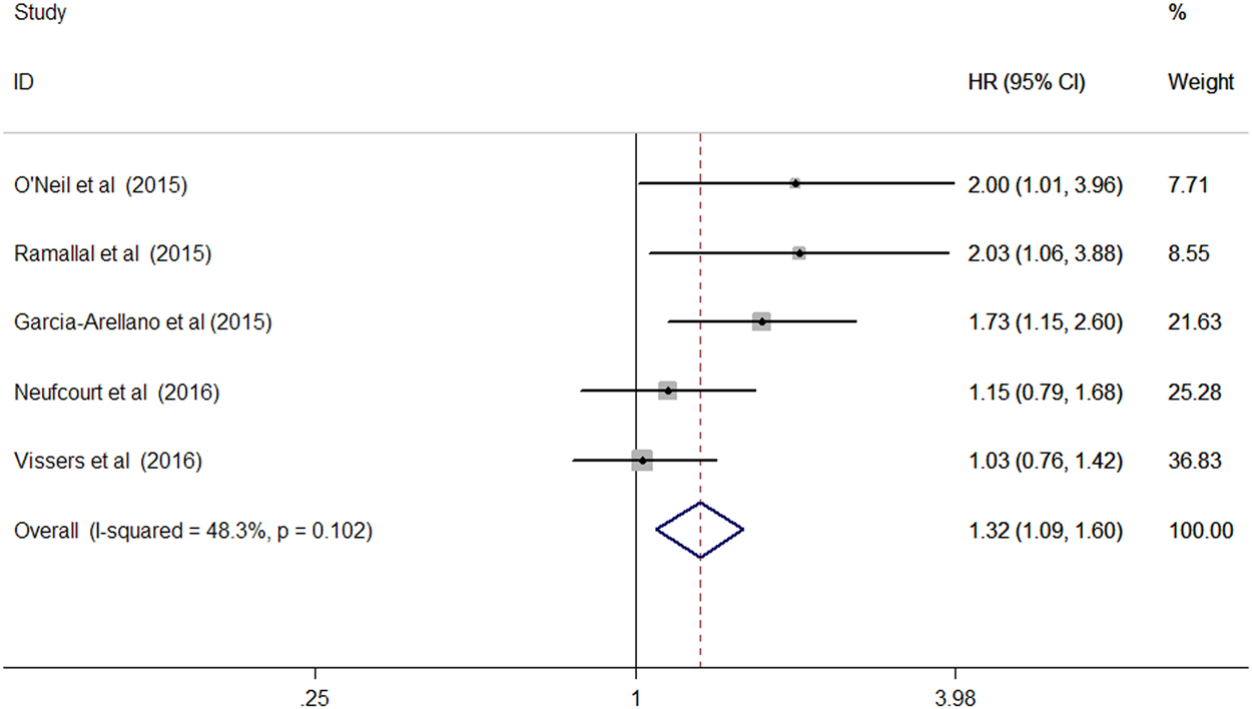
Forest plots showing HR with 95% CI of cardiovascular disease comparing the highest to the lowest dietary inflammatory index score.

Five studies^16–20^ reported 1,097 cardiovascular mortality events from 42,268 participants. As shown in Fig. 3, participants in the highest category of DII (strongly pro-inflammatory) was associated with increased risk of CVD (HR 1.32; 95% CI 1.09–1.60; I^2^ = 48.3%, *p* = 0.102) compared with those in the lowest DII category in a fixed-effect model. When we changed to a random effect model, the pooled HR for participants in the highest category of DII was 1.41 (95% CI 1.06–1.86) than those in the lowest DII category.

### Publication bias and sensitivity analyses

The small number of studies included in the individual outcome prevented us from conducting the publication bias using the Begg’s test and Egger’s test. Sensitivity analysis showed that individual study did not significantly impact on estimated overall effect size, indicating the reliability of our pooling results (data not shown).

## Discussion

The main finding of this meta-analysis indicates that more pro-inflammatory diets, as estimated by the higher DII score are independently associated with the increased risk of CVD and all-cause, cancer-related, cardiovascular mortality in the general population. Participants with the highest category of DII (maximal pro-inflammatory) led to 32% higher risk of CVD, 22% higher risk of all-cause mortality, 28% higher risk of total cancer-related mortality, and 24% higher risk of cardiovascular mortality. These findings strengthen the idea that there are incremental disadvantages to increase the pro-inflammatory dietary ingredients.

There are several approaches to assess the dietary quality. The Healthy Eating Index^23^, Alternate Healthy Eating Index^24^, and Dietary Approaches to Stop Hypertension Score^25^ are the most frequently used diet indexes. All of these healthy dietary patterns were associated with a decreased risk of all-cause mortality^24^. In contrast to above dietary score, DII represents a new dietary quality index that specifically focuses on the dietary inflammatory potential. DII is a literature-derived population-based dietary score to assess a total of 45 food parameters including various macronutrients, vitamins, minerals, flavonoid, and specific food items^11^. A higher DII score is representative of more pro-inflammatory diets. In practice, DII score was computed from dietary intake assessed using a validated food frequency questionnaire or 24-h recall dietary records. Findings from the Energy Balance Study indicated that the DII score was associated with above mentioned dietary indices^26^.

Studies that not satisfied the inclusion criteria for this meta-analysis also investigated the association of dietary inflammatory potential with CVD or mortality risk. In the European Prospective Investigation into Cancer and Nutrition study^27^, dietary inflammatory potential was assessed by means of an inflammatory score of the diet (ISD). Subjects with the highest ISD (more pro-inflammatory diet) had an HR of 1.42 for all-cause mortality, 1.89 for cardiovascular mortality, and 1.44 for cancer-related mortality. National Health and Nutrition Examination Survey (NHANES) III study^28^ showed that pro-inflammatory diet, as indicated by higher DII score, was associated with an increased risk of all-cause, CVD, all-cancer mortality among prediabetic patients. While in the NHANES study^29^, previously diagnosed CVD patients with the highest DII score significantly increased by 30% prevalence odds ratio of combined circulatory disorders, suggesting limiting pro-inflammatory diets may contribute to reduce the recurrence in these patients.

Chronic inflammation has a negative impact on the human body and health. The first step in the prevention and treatment of many chronic diseases is to follow a healthy dietary habit. Our meta-analysis reveals that limiting pro-inflammatory diets may be a healthy eating strategy to reduce CVD and mortality risk. An anti-inflammatory diet has beneficial effects on aging and health by slowing down telomere shortening^21^. In order to adopt a healthier diet, several anti-inflammatory foods including fruits, vegetables, fish or fish oil, walnuts, brown rice, and bulgur wheat should be part of the diet^30^. Moreover, avoid refined or processed foods and cut back on red meat or full-fat dairy foods are recommended.

This meta-analysis had some limitations. First, DII score was collected by self-report from 24-hour dietary recall or food frequency questionnaire, which carried an inherent degree of bias. Second, DII score was estimated at baseline and these data might change during the follow-up duration. However, adult dietary habits seem to relatively stable over time^31, 32^. Third, substantial heterogeneity was observed across studies pooling the mortality risk. Potential sources of heterogeneity included differences in the food items considered in the DII score, demographic characteristics, and follow-up duration. Fourth, the wide range of follow-up period (from 1.24 to 20.7 years) among the included studies was another limitation. However, sensitivity analyses showed no significant impact on the overall risk estimates when we excluded one study^14^ with the shortest follow-up duration. Finally, the majority of study participants were of European descent. Therefore, generalization of these findings to other ethnic populations should be taken with caution.

In conclusion, this meta-analysis suggests that more pro-inflammatory diets, as estimated by the DII score are independently associated with the increased risk of CVD and all-cause, cancer-related, cardiovascular mortality in the general population. However, whether promoting dietary patterns with low inflammatory potential could improve health status should be verified by more well-designed randomized controlled trials.

## Methods

### Search strategy

This meta-analysis followed the guidelines of the Preferred Reporting Items for Systematic Reviews and Meta-Analysis (PRISMA) statement. A comprehensive literature search was carried out in PubMed and Embase through February 2017 with the following search terms in various combinations: (inflammatory potential of diet OR dietary inflammatory index OR pro-inflammatory diet OR anti-inflammatory diet) AND (cardiovascular disease OR mortality OR death OR survival) AND (prospective OR longitudinal OR follow-up). In order to find additional studies, we also manually searched the reference lists of all relevant articles.

### Study selection

Studies were included if: (1) study participants were the general population; (2) study was prospective observational design; (3) the exposure of interest was the inflammatory potential of diets as estimated by DII score; and (4) reporting multivariable-adjusted risk estimates for the highest DII score (maximal pro-inflammatory diets) versus the lowest DII score (lowest pro-inflammatory diets) with respect to the all-cause, cardiovascular, cancer-related mortality, or CVD. Exclusion criteria were: (1) participants had a specific disease; (2) DII score as a continuous variable; (3) other inflammatory score of the diets as exposure; and (4) cross-sectional study, review or conference abstract.

### Data extraction and quality assessment

For each included study, information on first author’s surname, publication year, study location, study design, sample sizes, percentage of women, mean age or age range, dietary assessment method, DII score comparison, number of events, most fully adjusted risk estimate, follow-up duration, and adjustment for variables. The methodological quality of included studies was assessed by a nine-star Newcastle–Ottawa Scale (NOS) for cohort studies^33^. The NOS is categorized into selection, comparability, and ascertaining of outcome. Studies with ≥7 stars were defined as high quality. Data extraction and quality assessment were performed independently by two authors. Any discrepancies between two authors were resolved by consensus.

### Statistical analyses

All the analyses were conducted by the STATA software version 12.0 (Stata Corporation, College Station, Texas, USA). The multivariable-adjusted hazard risk ratio (HR) or odds ratio (OR) with 95% confidence interval (CI) was pooled for the highest versus the lowest DII score. The heterogeneity across studies was explored using the Cochrane Q and I^2^ statistic. If the Cochrane Q test ≤0.10 or I^2^ > 50%, the heterogeneity was considered as statistically significant^34^. Thus, a random effect model was selected to compute the summary effect; otherwise, a fixed-effect model was applied. Publication bias assessment with the Begg’s test^35^ and Egger’s test^36^ was planned when more than 10 studies were retrieved^37^. Sensitivity analysis was conducted by sequentially removed one study at a time.
